# A comparison of the transcriptome of *Drosophila melanogaster *in response to entomopathogenic fungus, ionizing radiation, starvation and cold shock

**DOI:** 10.1186/1471-2164-16-S13-S8

**Published:** 2015-12-16

**Authors:** Alexey Moskalev, Svetlana Zhikrivetskaya, George Krasnov, Mikhail Shaposhnikov, Ekaterina Proshkina, Dmitry Borisoglebsky, Anton Danilov, Darya Peregudova, Irina Sharapova, Eugenia Dobrovolskaya, Ilya Solovev, Nadezhda Zemskaya, Lyubov Shilova, Anastasia Snezhkina, Anna Kudryavtseva

**Affiliations:** 1 Laboratory of Molecular Radiobiology and Institute of Biology; Komi Science Center of Russian Academy of Sciences; Syktyvkar, Russian Federation; 2 Syktyvkar State University; Syktyvkar, Russian Federation; 3 Laboratory of genetics of aging and longevity, Moscow Institute of Physics and Technology, Dolgoprudny, Russian Federation; 4 Laboratory of Post-Genomic Research, Engelhardt Institute of Molecular Biology, Russian Academy of Sciences, Moscow, Russian Federation; 5 Institute of Mathematics and Computer Science, University of Latvia, Riga, Latvia

**Keywords:** Survival, Lifespan, Gene expression, *Drosophila melanogaster*, Entomopathogenic fungus, Ionizing radiation, Starvation, Cold shock

## Abstract

**Background:**

The molecular mechanisms that determine the organism's response to a variety of doses and modalities of stress factors are not well understood.

**Results:**

We studied effects of ionizing radiation (144, 360 and 864 Gy), entomopathogenic fungus (10 and 100 CFU), starvation (16 h), and cold shock (+4, 0 and -4°C) on an organism's viability indicators (survival and locomotor activity) and transcriptome changes in the *Drosophila melanogaster *model. All stress factors but cold shock resulted in a decrease of lifespan proportional to the dose of treatment. However, stress-factors affected locomotor activity without correlation with lifespan. Our data revealed both significant similarities and differences in differential gene expression and the activity of biological processes under the influence of stress factors.

**Conclusions:**

Studied doses of stress treatments deleteriously affect the organism's viability and lead to different changes of both general and specific cellular stress response mechanisms.

## Background

Gene expression changes underlie the organism's response to different types of stress factors. The detection of general and specific stress response genes may contribute to revealing the mechanisms of organism's adaptation to adverse conditions. In previous studies, the gene expression changes under normal *Drosophila *aging as well as after stress treatments including heat and cold shock, ionizing radiation exposure, oxidative stress (hyperoxia and hydrogen peroxide), heavy metal stress (cadmium, zinc, copper), and starvation were investigated using microarrays. It was shown that stress conditions led to enhanced transcriptional activity of general genes involved in free radical detoxification, heat shock response, mitochondrial unfolded protein response, immunity, circadian rhythm regulation, and reproduction. Additionally, each influence induces a set of specific changes in gene expression [1-4]. Perturbations in the activity of genes involved in development, stress, immune response and metabolism were found 24 h after impact of organic pollutant endosulfan [5]. The mild but noticeable effect on gene expression profiles in *Drosophila *was also found for reduced gravity [6]. Other studies showed that transcriptome differences in genes involved in metabolism, cell membrane composition, stress and immune response, and circadian rhythms determine the adaptation of *Drosophila *species and populations to environmental conditions, for example temperature [7,8].

Recent transcriptome studies showed that a broad range of stress treatments (e.g. cold, heat, caffeine, paraquat, rotenone, copper, zinc, cadmium, formaldehyde, dioxin, and low doses of ionizing radiation) differentially affects expression of both general and specific stress response genes [9-11]. General stress responses in *Drosophila melanogaster *include the activation of genes of cell cycle control, formation of gametes, circadian rhythms, splicing, proteolysis, and various aspects of metabolism [10], as well as genes that encode lysozymes, cytochrome P450s, and mitochondrial components mt:ATPase6, mt:CoI, mt:CoIII [11]. Most stress factors strongly down-regulate genes responsible for cell respiration, cell-cell communication, and various aspects of metabolism, immune response, and response to light stimuli [10], egg-shell, yolk, and seminal fluid proteins [11]. Using microarray analysis and transcriptome sequencing, it was also shown that age-associated changes in levels of gene expression shared features with stress-response, such as oxidative stress [2] and ecopollutants [10]. Thus, the advancements in transcriptomics have allowed for the possibility to study molecular mechanisms underlying the organism's response to various stress factors.

This paper aims to reveal gene pathways involved in the response to various stress types and to study the molecular mechanisms determining the organism's reactions to stress factors. We analyzed the effects of entomopathogenic fungus, ionizing radiation, starvation, and cold shock on survival, age dynamics of locomotor activity, expression of green fluorescent protein (GFP) reporter of stress response genes (i.e. *Hsp22 *and *Hsp70*, *Defensin*, *Drosomycin*, *Metchnikowin*, *GstD1*, *D-GADD45*, and *Stat92E*), and transcriptome changes in the model animal *Drosophila melanogaster*. All stress treatments but cold shock decreased lifespan in proportion to the dose of treatment. The effects on locomotor activity were not correlated with lifespan effects. We observed both significant similarities and differences in differential gene expression and activities of biological processes.

## Results

### Analysis of survival and locomotor activity

**Figure 1:**
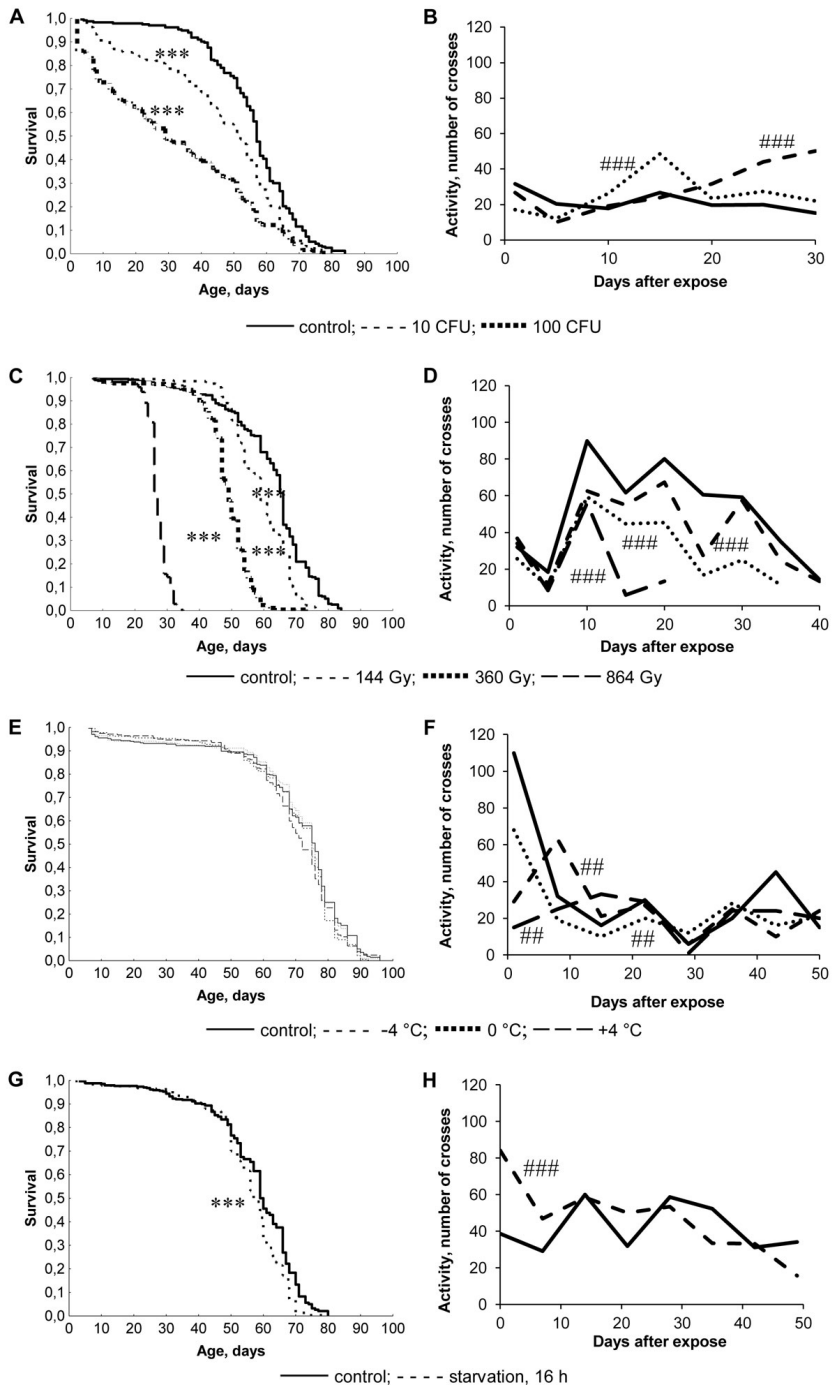
**Effect on survival functions (A, C, E, G) and locomotor activity (B, D, F, H) of male *Drosophila*; (A, B) fungus, (C, D) radiation, (E, F) cold shock, and (G, H) starvation**. Survival combines 2-3 repetitions of experiments, and locomotor activity shows average sensor response in 3 min per 30 flies. ***p < 0.001 - Kolmogorov-Smirnov test. ^###^p < 0.001 χ^2 ^test

To evaluate the stress effects at the organism level, we analyzed survival and locomotor activity. The treatment of flies with 10 and 100 CFU (colony-forming units per individual) of entomopathogenic fungus, ionizing radiation in doses of 144, 360 and 854 Gy, and 16 hour starvation decreased lifespan (Table 1 Figures 1A, C, G). In the case of entomopathogenic fungus and ionizing radiation, the effect was proportional to the dose of stress factor, which corresponds to published data [12,13]. No statistically significant effects on lifespan were observed after cold shock (Table 1 and Figure 1E).

**Table 1:** Effect of stress factors on survival functions of male imago *Drosophila melanogaster*.

Factor	Dose	M (days)	dM	90% (days)	d90%	n (exp.)	n
Entomopathogenic Fungus	control	57		71		3	233
	
	10 CFU	51.5***	-9.6%	66^#^	-7%	3	216
	
	100 CFU	29***	-49.1%	65^###^	-8.5%	3	254

Ionizing Radiation	control	66		77		2	315
	
	144 Gy	59***	-10.6%	69^###^	-10.4%	2	310
	
	360 Gy	49***	-25.8%	56^###^	-27.3%	2	286
	
	864 Gy	27***	-59.1%	32^###^	-58.4%	2	357

Starvation	control	60		71		2	338
	
	16 h	57**	-4.3%	68^###^	-4.2%	2	367

Cold Shock	control	59		71		2	285
	
	+4°C	59	0	73	+2.8%	2	327
	
	0°C	57	-3.4%	70	-1.4%	2	308
	
	-4°C	59	0	70	-1.4%	2	297

**Notes: **M - median lifespan, 90% - age of 90% mortality, dM and d90% - difference between control and experiment, n (exp.) - number of repetitions of experiment, n - total number of analysed flies.

**p < 0.01, ***p < 0.001 - Mantel-Cox test

#p < 0.05, ###p < 0.001 - Wang-Allison test

The treatment with entomopathogenic fungus, ionizing radiation, and cold shock decreased locomotor activity 1-5 days after treatment (Figures 1B, D, and 1F). However, starvation increased locomotor activity up to 25 days after treatment (Figure 1C). Ten days after treatment, locomotor activity declined in irradiated flies (Figure 1D) and increased in flies treated with entomopathogenic fungus (Figure 1B). Cold shock resulted in various effects on locomotor activity (Figure 1F). Thus, we didn't observe correlation between the effects of stress-factors on lifespan and locomotor activity. The locomotor activity seems not to be crucial for the organism's survival in normal conditions after stress treatment. The obtained results suggest that lifespan is an integral indicator of the organism's viability, and does not reflect the condition of the particular indicators, such as locomotor activity.

### Analysis of GFP reporter expression

**Figure 2:**
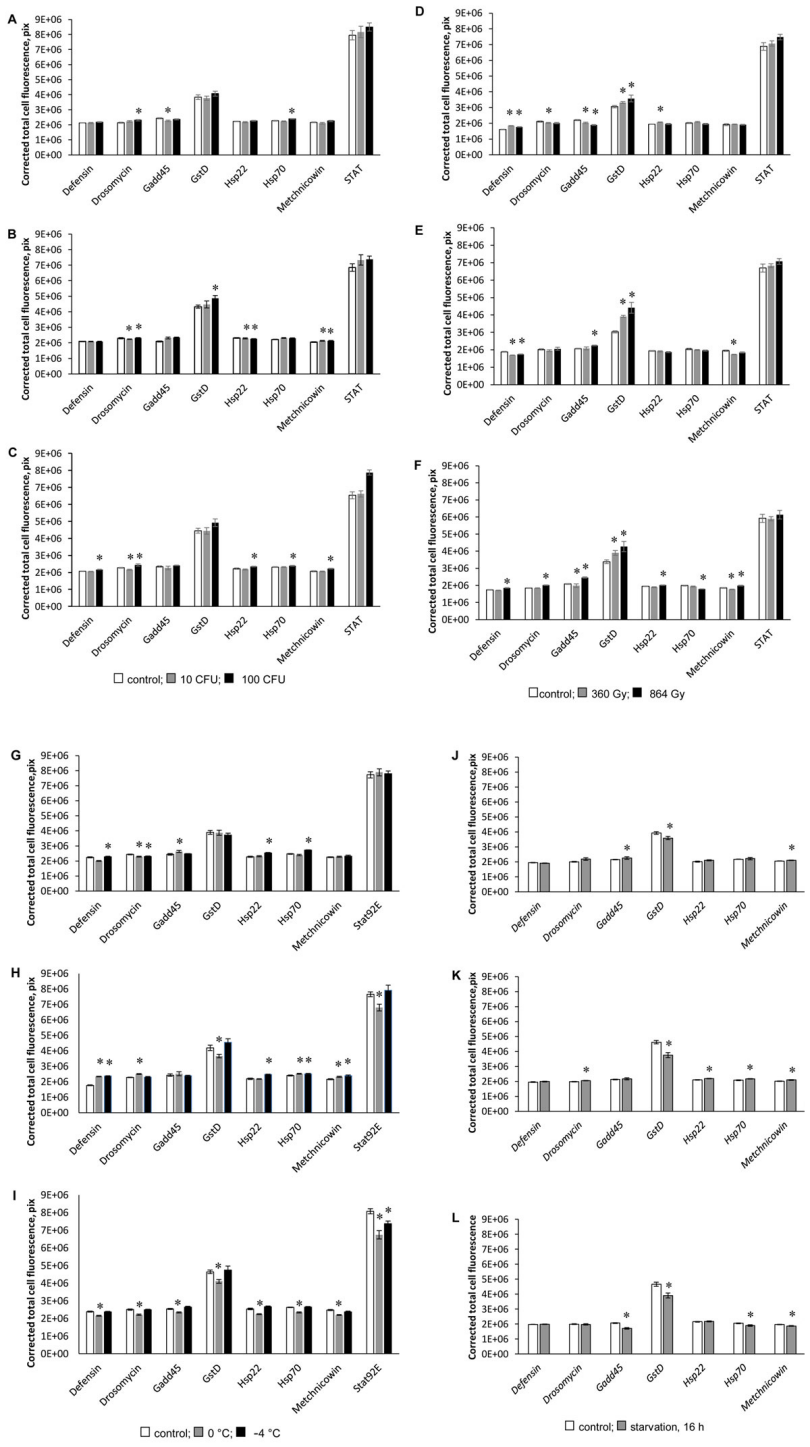
**Effect on expression of GFP reporters of stress response genes; (A, B, C) fungus, (D, E, F) radiation, (G, H, I) cold shock, and (J, K, L) starvation**. Expression of GFP was measured (A, D, G, J) 1 day, (E, H, K) 2 days, (F, I, L) 3 days, (B) 4 days, and (C) 5 days post-treatment. * p < 0.05, t-test.

We studied the effects of stress factors on expression levels of GFP reporters of 8 stress response genes (*Hsp22*, *Hsp70*, *Defensin, Drosomycin, Metchnikowin, GstD1, D-GADD45*, and *Stat92E*) at different times after exposure to different doses (Table 2 Figure 2). The expression level of GFP reporters of stress response genes depended on multiple factors, including dose of treatment and time period post-treatment. The observed effects are consistent with numerous published studies, which were performed on *Drosophila *and other models [2,14-25]. Since the expression level of GFP-reporters directly reflects the activity of the genes of interest, these results were used to validate RNA sequencing data.

**Table 2:** Effect of stress factors on expression of GFP reporters of stress response genes.

	Entomopathogenic Fungus						Ionizing Radiation						Cold Shock						Starvation		
	
Gene of Interest	1 day		4 days		5 days		1 day		2 days		3 days		1 day		2 days		3 days		1 day	2 days	3 days
	
	10 CFU	100 CFU	10 CFU	100 CFU	10 CFU	100 CFU	360 Gy	864 Gy	360 Gy	864 Gy	360 Gy	864 Gy	0°C	-4°C	0°C	-4°C	0°C	-4°C	16 h	16 h	16 h
*Defensin*						↑	↑	↑	↓	↓		↑	↓		↑	↑	↓				

*Drosomycin*		↑			↓	↑	↓					↑	↓	↓	↑		↓		↑	↑	

*Gadd45*	↓		↑	↑			↓	↓		↑	↓	↑	↑				↓		↑		↓

*GstD1*				↑			↑	↑	↑	↑	↑	↑			↓		↓		↓	↓	↓

*Hsp22*						↑	↑					↑		↑		↑	↓			↑	

*Hsp70*		↑	↑	↑		↑						↓		↑	↑	↑	↓			↑	↓

*Metchnicowin*			↑	↑		↑			↓		↓	↑			↑	↑	↓		↑	↑	↓

*STAT*						↑									↓		↓	↓	-	-	-

**Notes: **↑ - up-regulation; ↓ - down-regulation; empty cell - no differential expression; - (dash) - no analysis performed.

### Analysis of the transcriptome

**Figure 3:**
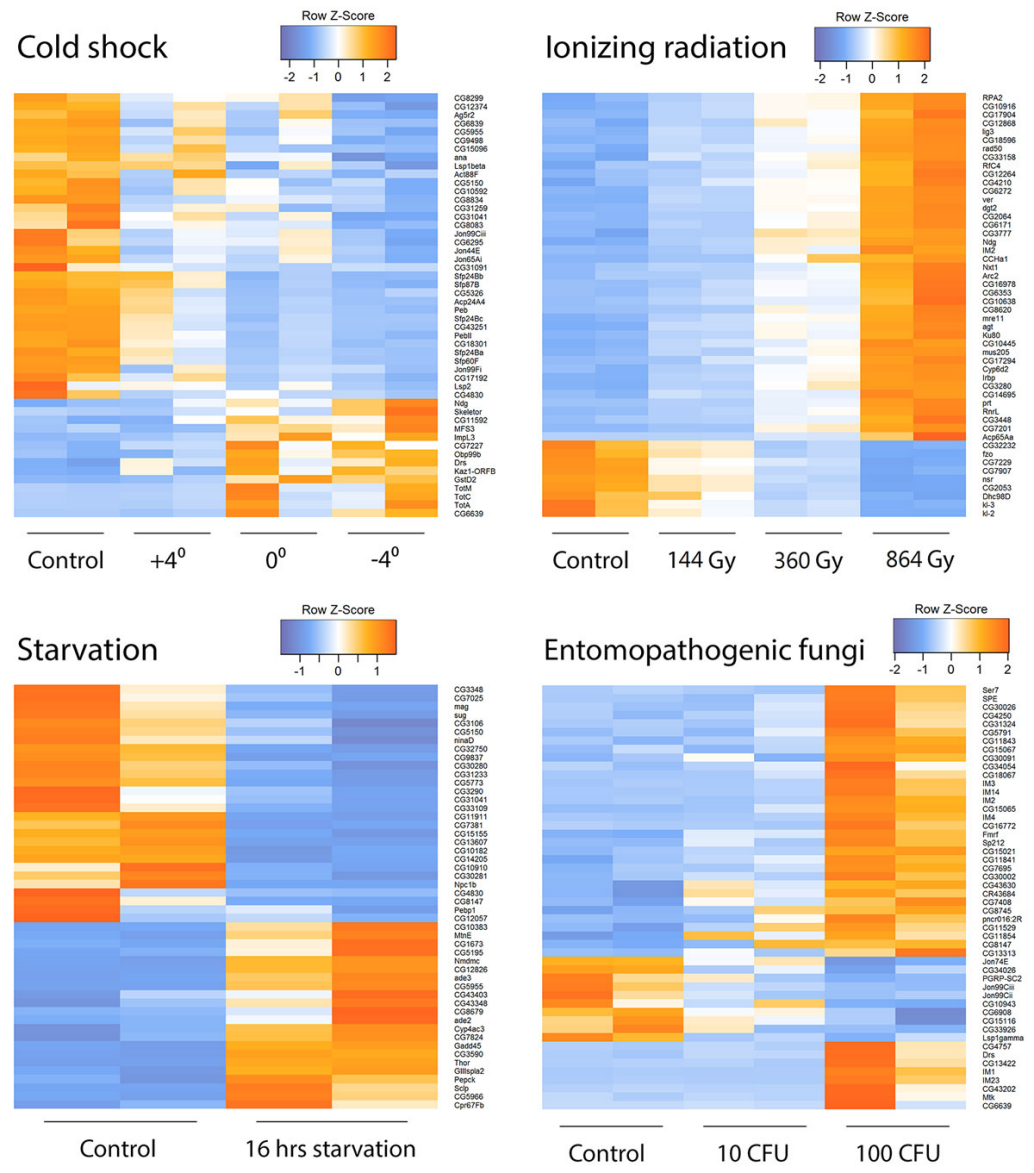
**Top 50 differentially expressed genes (entomopathogenic fungus, ionizing radiation exposure, cold shock, and starvation)**.

We performed transcriptome profiling of *Drosophila melanogaster *for 4 stress factors in 9 treatments, namely, entomopathogenic fungus (10 and 100 CFU), ionizing radiation (144, 360 and 864 Gy), starvation (16 h), and cold shock (-4°C, 0°C and +4°C), see Figure 3. Differentially expressed genes were identified using adjusted p-value (FDR), which provides more accurate results in comparison to regular p-values. Using adjusted p-values < 0.05, the following transcripts were differentially expressed.

Entomopathogenic fungus at a dose of 10 CFU down-regulated 135 genes and up-regulated 133 genes, whereas fungus of 100 CFU down-regulated 288 genes and up-regulated 363 genes (Additional file 1 Table S1). The generalized linear model (GLM) approximation approach allowed us to identify 90 down-regulated and 161 up-regulated genes, with CFU doses affecting the expression of genes. Eight of the top 10 (and 19 of the top the 50) up-regulated genes involved a defense response against bacteria and fungus, including immune response and Toll signaling pathway activation. Genes of antimicrobial peptides *Metchnikowin*, *Drosomycin*, *Drosocin*, and *TotM *genes (4-fold up-regulated) are among these genes, and this is concordant with previously published results [26]. The turandot family member *TotC *shows a 2-fold up-regulation, while other members do not demonstrate expression alterations. We observed 6 immune-induced peptides (i.e. IM1, 2, 3, 4, 14, 23) among the top 20 up-regulated genes. These peptides participate in the innate immunity response. Down-regulated gene sets are enriched with monosaccharide metabolic process Gene Ontology terms (8 of top 50 down-regulated genes). Proteolysis, namely the serine-type peptidase activity GO term, is enriched with both top up-regulated (9 of top 50) and down-regulated (8 of top 50) genes.

**Figure 4:**
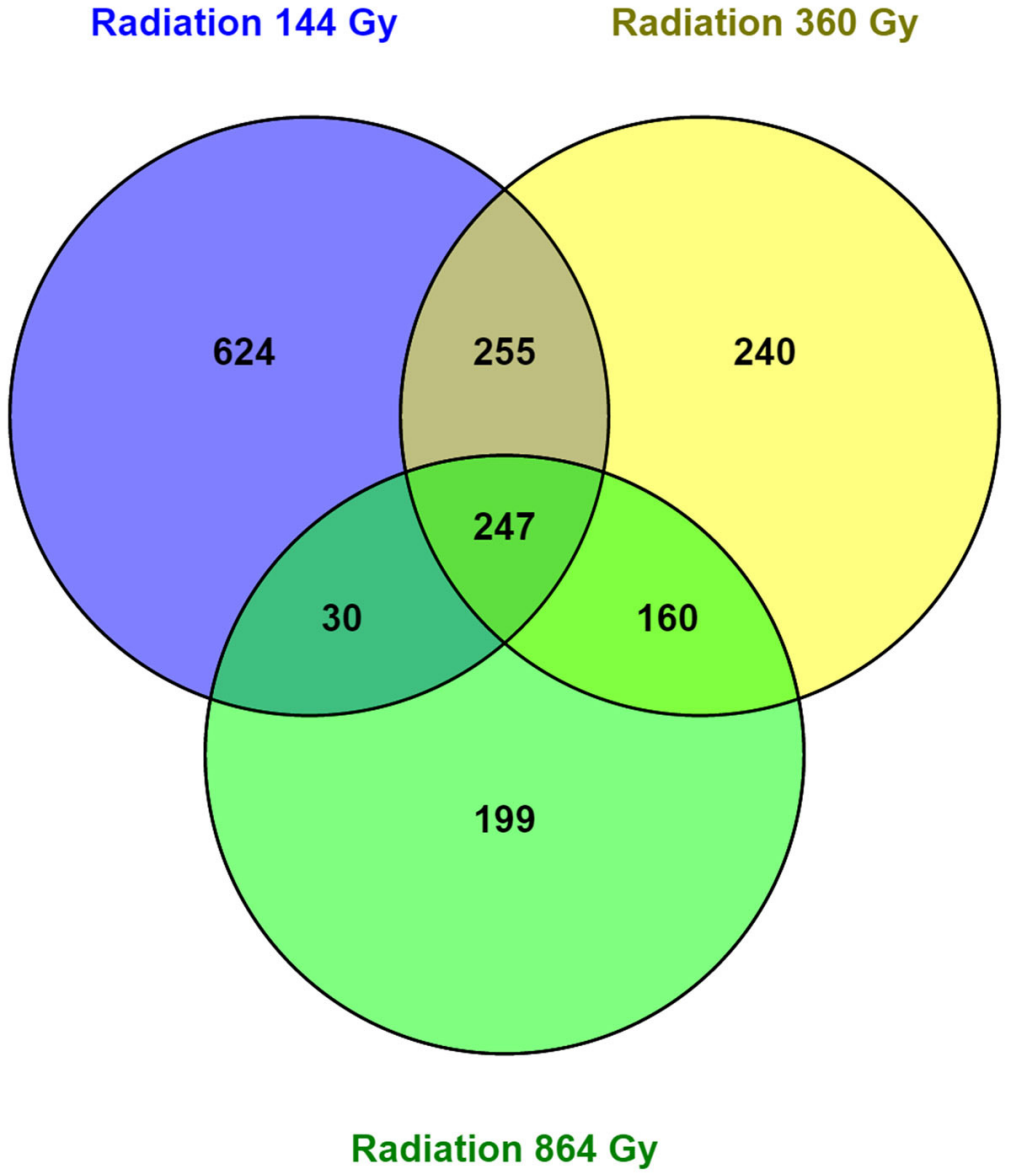
**Diagram representing the quantity of shared genes between the different dose radiation exposures**.

**Figure 5:**
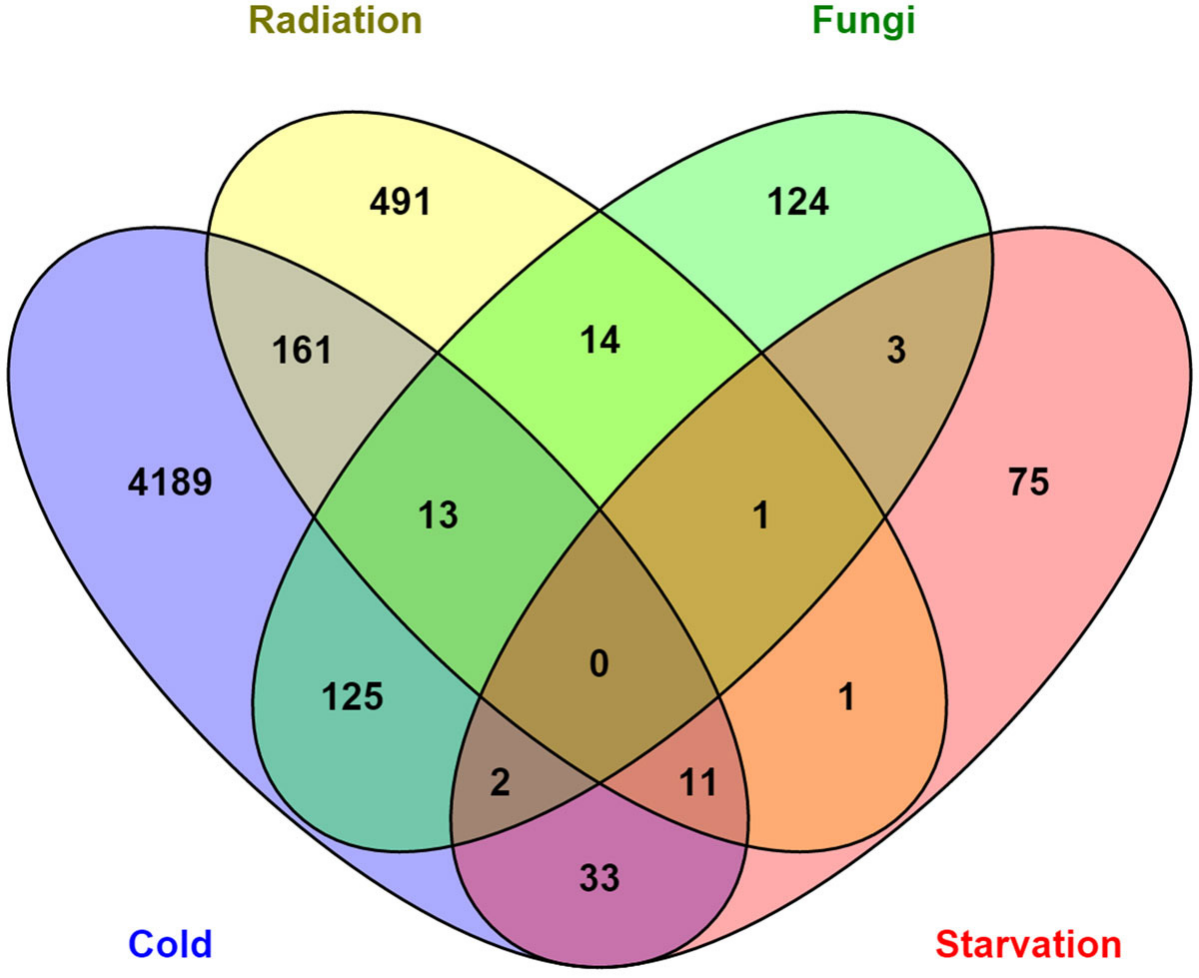
**Diagram representing the quantity of shared genes between the different treatments**.

Dose of ionizing radiation affects gene expression (Figure 4). Ionizing radiation of 144 Gy resulted in 670 down- and 486 up-regulated genes, 360 Gy resulted in 466 down- and 436 up-regulated genes, and 864 Gy resulted 330 down- and 306 up-regulated genes. Starvation resulted in 59 down- and 67 up-regulated genes. Cold stress resulted in the largest number of differentially expressed genes: 5790, 2803, and 4802 down- and 151, 312, and 115 up-regulated genes for -4°C, 0°C and +4°C, correspondingly (Additional file 1 Table S1). One hundred genes were differentially expressed for five or more treatments, and 5203 genes were differentially expressed for two or more treatments with 647 differentially expressed genes for more than one type of stress (fungus, irradiation, starvation, or cold), see Figure 5.

**Figure 6:**
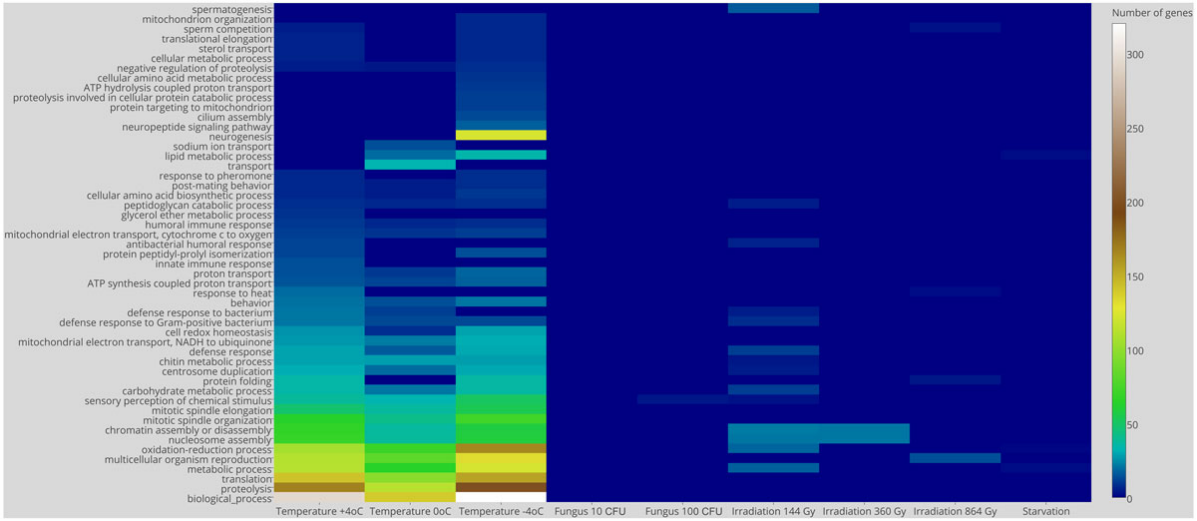
**Heat map of down-regulated biological processes based on Gene Ontology IDs**.

**Figure 7:**
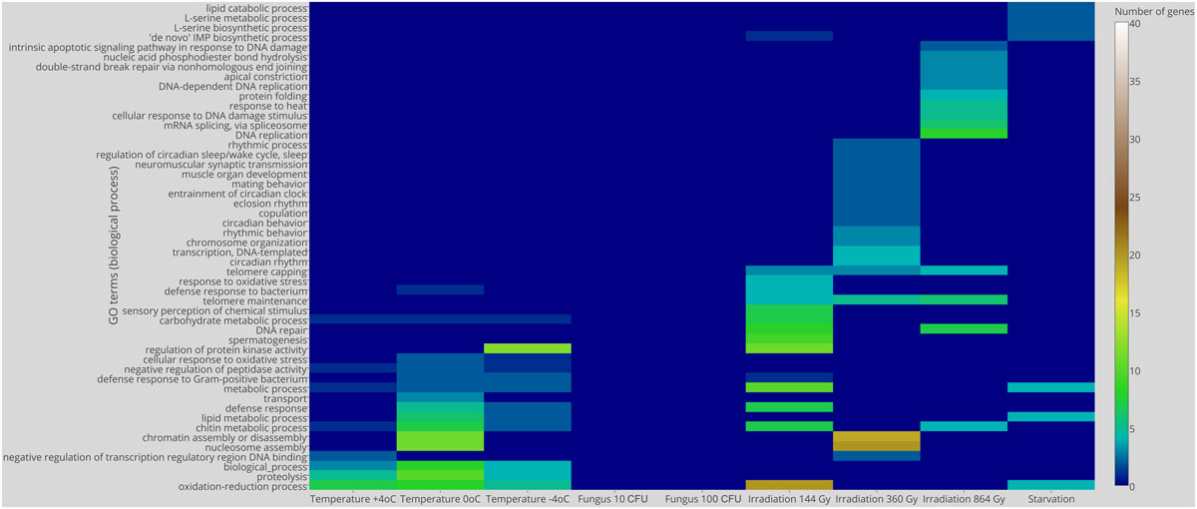
**Heat map of up-regulated biological processes based on Gene Ontology IDs**.

Gene set enrichment analysis (GSEA) using Gene Ontology (GO) Biological Processes ontology provided the following observations. Twenty two biological processes shared down-regulated genes affected by 5 or more treatments (Figure 6). The oxidation-reduction process was affected by all treatments except entomopathogenic fungus and ionizing irradiation of 360 and 864 Gy, and up-regulated by all treatments except entomopathogenic fungus and ionizing irradiation. Metabolic process and chitin metabolic process were among the most enriched by down- and up-regulated genes that were differentially expressed in many treatments (Figure 7).

In addition to biological processes affected by the many treatments, we observed biological processes affected by a single stress factor. Starvation affects the 'de novo' IMP biosynthetic process, cellular amino acid biosynthetic process (including L-serine biosynthetic and metabolic process), determination of adult lifespan, lipid metabolic process with lipid metabolic process enriched by down-regulated genes.

Ionizing radiation from 144 to 864 Gy up-regulated genes of 39 biological processes. Two biological processes (telomere maintenance and telomere capping) were up-regulated by 144 and 864 Gy radiation. Ionizing radiation from 144 to 864 Gy down-regulated genes of 47 processes, 2 of them (sperm individualization and mRNA processing) were affected by 360 and 864 Gy. Due to the sex constraint, all female-specific processes were excluded from the analysis.

Cold stress of -4°C, 0°C and +4°C up-regulated genes of 35 biological processes. Six of them were up-regulated by all three cold stress treatments, and 3 processes were up-regulated by -4°C and 0°C (lipid metabolic process, defense response, defense response to Gram-positive bacterium). Cold stress of -4°C, 0°C and +4°C down-regulates genes of 35 biological processes (Figure 6). Thirty two of them were common for all treatments and mostly related to metabolic processes and post-mating behavior. It is worth noting that the -4°C treatment down-regulated mitochondrion organization and oxidation-reduction processes. Previously, oxidative stress was reported for chilling-induced stress in mume fruit [27], and this, as well as cold, was treated as an abiotic factor that increases the number of free radicals [28,29]. These observations, however, were made for plants.

**Figure 8:**
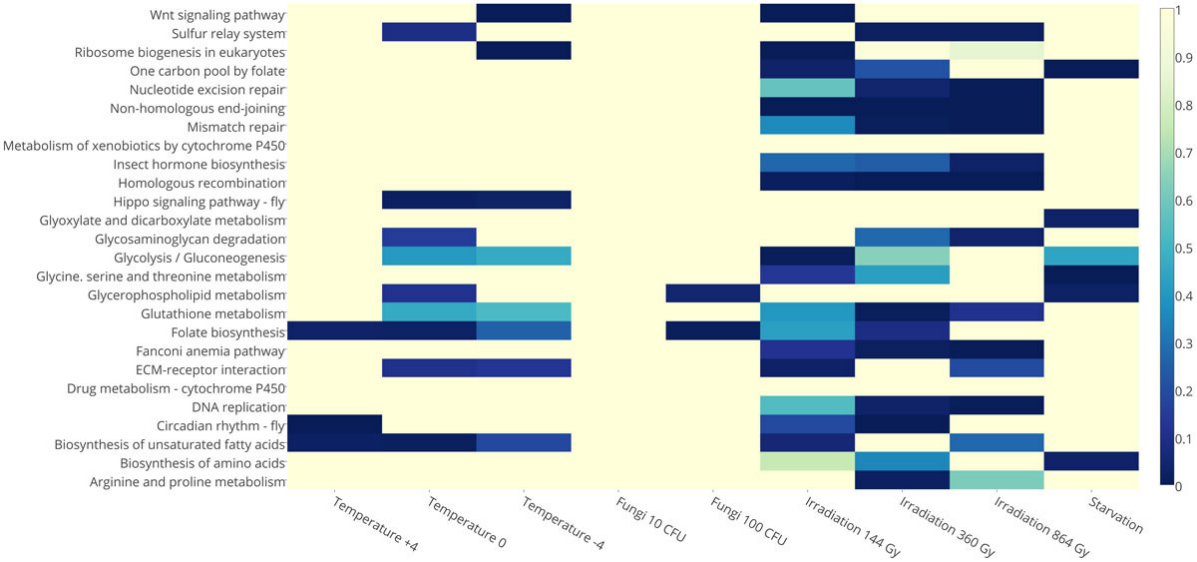
**Heat map of up-regulated molecular pathways based on KEGG analysis IDs, color-coded by the number of genes**.

KEGG pathway ontology was used in GSEA. Twenty seven pathways were differentially expressed by two or more treatments, with 14 up- and 13 down-regulated, see Figure 8, 9 correspondingly. Most of these pathways are related to metabolism or biosynthesis of amino acids and other substances; others, i.e. homologous recombination, non-homologous end-joining and Wnt signaling pathway, and circadian rhythm were up-regulated; ribosome, pentose and glucuronate interconversions were down-regulated. Seven KEGG pathways were down-regulated by entomopathogenic fungus 100 CFU, two of which are related to metabolic processes (fructose, mannose, pentose and glucoronate) and are also down-regulated in response to 10 CFU treatment. Two KEGG pathways were up-regulated by entomopathogenic fungus 100 CFU: folate biosynthesis and glycerophospholipid metabolism. Eight KEGG pathways were differentially expressed by starvation, with metabolism of amino acids and other substances (e.g. glycerophospholipids) being up-regulated, and protein processing in endoplasmic reticulum, folate biosynthesis and alpha-Linolenic acid metabolism pathways being down-regulated. These changes due to starvation may be explained by the priority on metabolism of key molecules and the smaller intensity of other processes. Cold stress down-regulated one KEGG pathway under all examined temperature conditions - "one carbon pool by folate". Cold stress down-regulated many pathways, most of these were related to metabolism of substances, including, amino acids, folate, pyruvate and carbone, and ribosome function. Cold stress of -4°C down-regulated more pathways than other temperature treatments, and in addition to those already mentioned, it down-regulated RNA polymerase, protein export pathways, and ribosome functions. All radiation doses up-regulated genes of non-homologous end-joining and homologous recombination KEGG pathways. GO Molecular Function ontology was used in GSEA. Five or more treatments down-regulated DNA binding, N-acetylmuramoyl-L-alanine amidase activity, electron carrier activity, odorant binding, and peptidoglycan binding. Most of the treatments up-regulated DNA binding, chitin binding, serine-type endopeptidase activity, serine-type endopeptidase inhibitor activity, and triglyceride lipase activity.

**Figure 9:**
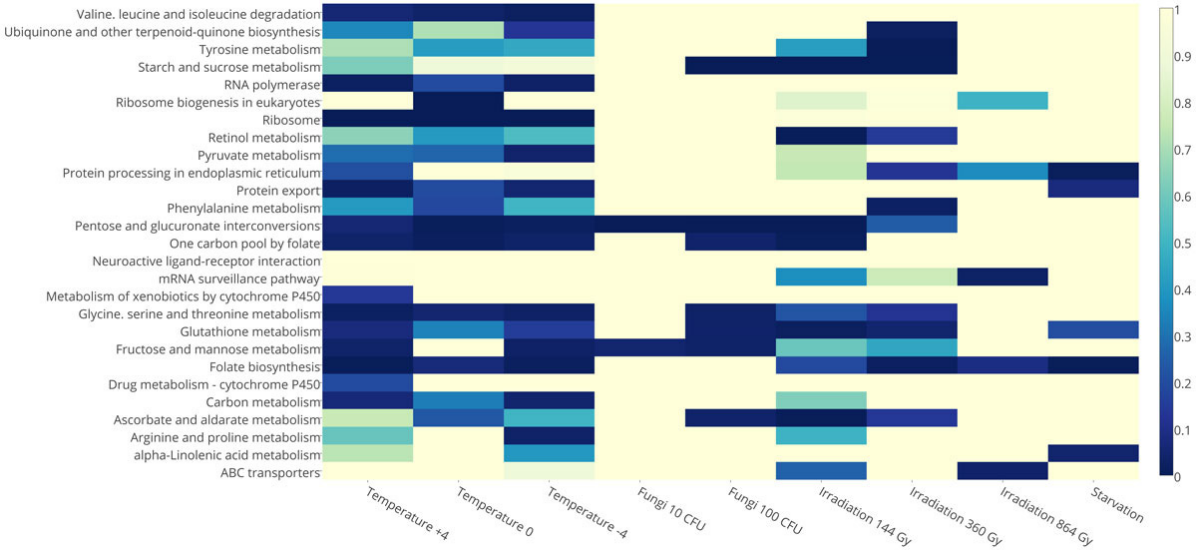
**Heat map of down-regulated molecular pathways based on KEGG analysis IDs, color-coded by the number of genes**.

The analysis of radiation doses showed genes with direct and inverse proportionality between dose and expression. Exposure to ionizing radiation led to dose-dependent up-regulation of hundreds of genes, with infrequent and weaker down-regulation. Whereas top down-regulated genes are related to GO Biological Processes (i.e. microtubule system and spermatogenesis), top up-regulated genes are mainly related to DNA repair and replication, telomere maintenance and capping. Cold shock was followed by dose-dependent down-regulation of thousands of genes, and a much smaller number up-regulated genes. Some of the down-regulated genes enrich proteolysis and metabolic process. Expression of 9 genes changed in proportion to the number of CFU of entomopathogenic fungus, with 4 genes down-regulated (*CG33926*, *Obp99b*, *CG15116*, *CR34335*), and 5 up-regulated (*IM1*, *CG4757*, *CG12057*, *Mtk*, *CG6639*). Genes of 3 biological processes are enriched (p < 0.05), namely, response to stimulus, response to stress, and innate immune response.

We observed changes in locomotor activity that are proportional to changes in expression of 82 genes induced by cold shock. Six of them show inverse proportionality (*CG8105*, *CG11854*, *Cpr67Fb*, *CG32694*, *tim*, *CG18530*), and 76 genes show direct proportionality.

## Discussion

Here we showed that all stress factors but cold shock have deleterious effects on lifespan, which are proportional to dose. However, we didn't observe a correlation between the effects of stress factors on lifespan and locomotor activity. Harmful effects on lifespan were accompanied by changes at the level of gene transcription. Differential expression analysis revealed both significant similarities and differences in gene expression and the activity of biological processes under the influence of entomopathogenic fungus, ionizing radiation, starvation, and cold shock. Stress response on the molecular level is dose-specific. Dose may influence the effects of ionizing radiation [30]. Low radiation (20-75 cGy) produces minimal cluster damages and breaks of double-stranded DNA, with the main effect related to the induction of active oxygen forms [31]. The main negative effect of higher doses of radiation is induced by damages of macromolecular structure. In conditions of cold shock, freezing temperatures (-4°C and 0°C) have a less harmful effect than the temperature between the comfort zone and freezing (+4°C) [32-34].

Six or more treatments differentially expressed 15 genes, with 4 of them affected by other treatments in addition to radiation and cold shock. The *Hsp22 *gene was down-regulated by cold shock of +4°C and up-regulated by radiation from 144 to 854 Gy and cold shock of 0°C and -4°C. According to expression analysis of GFP reporters, this gene was up-regulated one day post-treatment of cold shock or 864 Gy radiation. We may conclude that data from transcriptome sequencing do not contradict the results from the GFP analysis. In addition to that, *Hsp70Aa *was down-regulated by cold shock of +4°C and 0°C and 360 Gy irradiation, though GFP analysis indicated up-regulation of this protein one day post-treatment. This may indicate a different stress response speed for *Hsp22 *and *Hsp70 *heat shock proteins. *CG6295 *was down-regulated by 7 treatments of 3 stress factors, ionizing radiation, starvation, and cold shock. Its protein is a lipase generating free fatty acid from dietary lipid for absorption [35]. Aging induces adaptive metabolic response including down-regulating expression of this gene [36], as well as oxidative shock and radiation of 20 cGy [10], which may indicate its important role in stress response; perhaps energy metabolism is one of the key pathways affected by all types of stress.

The *CG7381, CG14219, Npc1b, CG11911 *genes were down-regulated by radiation, starvation, and cold shock. While these effects of starvation and cold shock may be explained by direct influence over metabolic process, radiation should be reviewed in greater detail. No functions are described for *CG7381*, *CG14219*, and *CG11911 *[37]. *Npc1b *(Niemann-Pick type C1) has few well studied orthologs and produces a protein utilised in sterol absorption and intracellular sterol trafficking [38]. Reduction of sterols due to starvation and irradiation may down-regulate expression of this gene [39] and determine similarity of metabolic processes in responses to different stress factors.

*CG15068 *was down-regulated by cold shock and up-regulated by ionizing radiation. GML approximation showed *CG15068 *expression alterations depending on radiation dose (p < 0.05). The exact function of the *CG15068 *gene is not clear, but some studies identify it as the gene coding immune response induced protein [40]. Low dose radiation exposure activates innate immunity through Toll pathway activation [18]. Such effects were probably caused by *CG15068 *gene expression changes.

Cold shock resulted in overexpression of five genes: *GstD2*, *TotM*, *TotC*, *TotA*, and *CG6639*. Three of them - *TotA*, *TotC*, *TotM*, and *TotX *(which are both down- and up-regulated to a lesser extent) belong to Turandot family of stress-induced humoral factors associated with resistance to the lethal effects of high temperature. A total of 8 Turandot genes are found in the *Drosophila melanogaster *genome. All of them were earlier shown to be up-regulated by bacterial infection, heat shock, mechanical pressure, dehydration, N, N'-dimethyl-4, 4'-bipyridinium dichloride (Paraquat), and ultraviolet [41]. Flies with up-regulated *TotA *show prolonged survival and retain normal activity at otherwise lethal temperatures [42]. Strikingly, *TotA *is also up-regulated during metamorphosis and at high age [42]. Recently, *Drosophila TotA *was found to be responsible for tolerance to methylmercury, an environmental neurotoxicant that targets the developing nervous system [43], whereas *TotM *promotes immunity against sexually transmitted fungal infections [26]. Using GLM approximation, we attempted to find genes that show expression alterations depending on cold shock strength. One hundred eleven genes were down-regulated, whereas only 6 genes were up-regulated by cold shock (Figure 3). The most top down-regulated genes belong to serine endopeptidases (18 of top 50 genes), and the most over-represented down-regulated pathway here is proteolysis.

As for cold shock, endopeptidases was the most down-regulated gene family (17 of top 50 genes) for 16 hour starvation. Among them, serine proteases takes a major place (13 of 17 genes). In addition, the following classes were highly enriched with down-regulated genes: exopeptidases, alkaline phosphatases, and genes participating in lipid transport. In contrast, top up-regulated genes under fasting conditions were mainly associated with aminoacid metabolism, biosynthesis of organonutrient compounds (including rubinucleoside), transaminase and lipase activity, and metal ion homeostasis. There were no differentially expressed genes among the shock-associated Turandot family. Thus, 16 hour starvation mainly led to down-regulation of proteolysis-associated genes and up-regulation of genes participating in biosynthesis of organonutrient compounds. However, 16 hours is a short period of time. In the long-term perspective (several days), starvation could lead to activation of proteolysis in order to provide supplements with needed nutrients. This was shown for various organisms [44-46].

**Figure 10:**
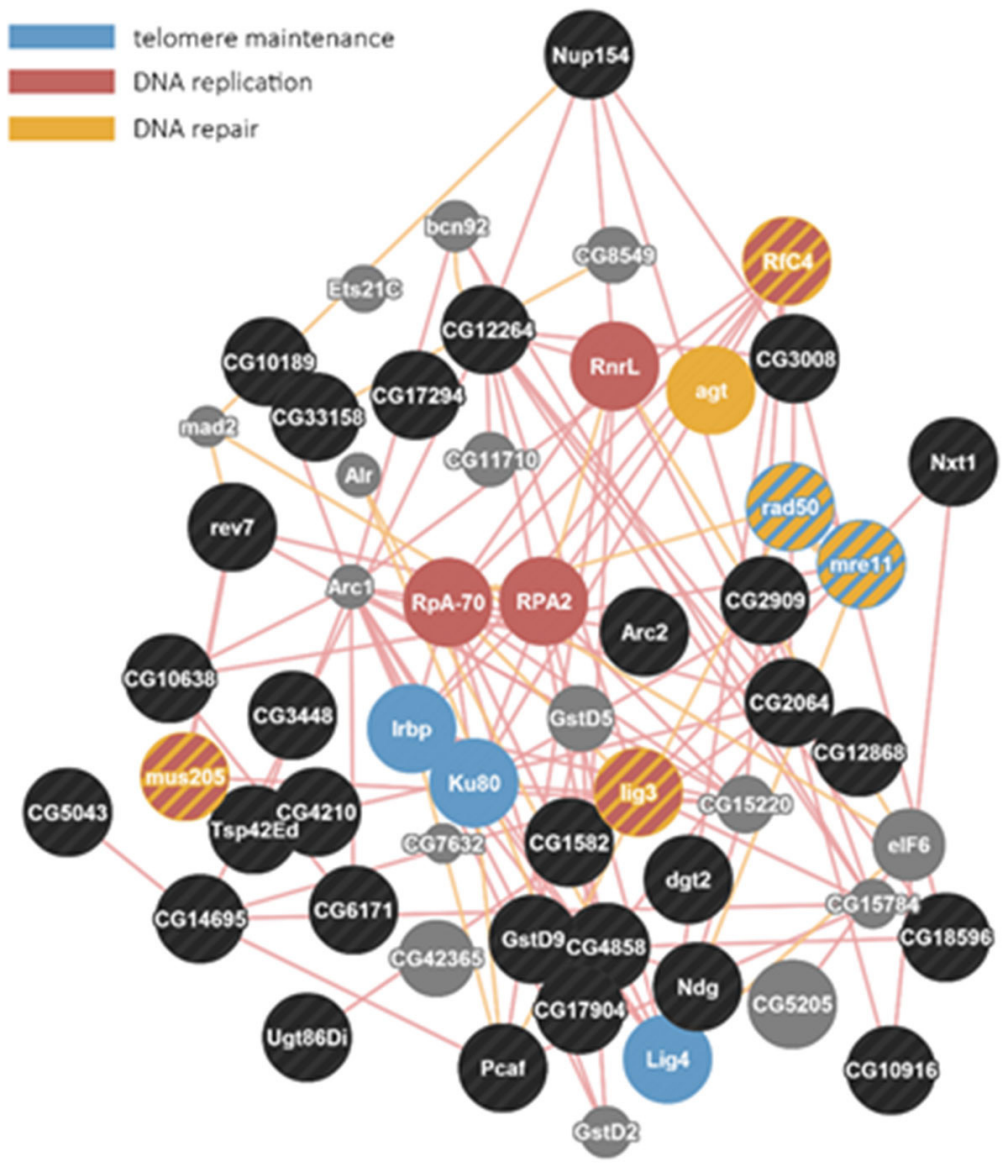
**Fragment of protein interaction network for 50 genes with dose-dependent up-regulation after exposure to ionizing radiation**. Protein involved in DNA replication and repair, telomere maintenance are marked with colors.

The interaction network for radiation is shown in Figure 10, gamma-ray exposure led to only a slight (1.5-2-fold) up-regulation of the *TotA *gene, whereas other members of the Turandot family did not show significant alterations. Complete dose-dependent up-regulation of *CG7201*, *Acp65Aa*, *CG32625*, *CG14545 *were also observed. Expression of these genes was very low or nearly absent in the control group (less than 0.02-1.0 reads per million, cpm) and is dramatically up-regulated by ionizing radiation (5-10 cpm in the group with maximal gamma-ray dose).

*Metchnikowin *(*Mtk*) was observed as the most up-regulated gene by fungal treatment. This is a 26 aa proline-rich peptide with antibacterial and antifungal properties first discovered by Jules Hoffman and Elena Levashina in 1995 and named after the Russian immunologist Ilya Mechnikov [47]. Induction of metchnikowin expression after immune challenge can be mediated either by the Toll pathway or by the imd (Immune Deficiency) gene product [48]. The up-regulated genes participate in Toll signalling, whereas *imd *expression was unaffected. This suggests the first mechanism plays a primary role here.

*Drosomycin*, the second top up-regulated gene, is an inducible antifungal peptide of 44 residues. Drosomycin exhibits a narrow antimicrobial spectrum and is only active against some filamentous fungus [49,50]. Like Metchnikowin, the systemic expression of Drosomycin is regulated by the Toll pathway present in the fat body, whereas inducible local expression in the respiratory tract is controlled by the imd pathway [49]. Homologues of Drosomycin with antifungal activity are found in humans [51].

Some changes in transcription may be related to defense and adaptation (e.g. activation of reparation system and antioxidative processes, changes in behaviour and sensitivity of signal pathways), and other changes are related to dysfunction of cell systems (e.g. problems in biosynthesis processes and substances metabolism, in circadian rhythm and balance between oxidation and reduction processes). We mostly observed stress-induced lifespan reduction though *Drosophila *defense mechanisms, which were activated with up-regulation of defensive pathways related to response to oxidative stress, nucleosome assembly, response to DNA damage, DNA repair, and telomere maintenance.

GSEA with GO Biological Process ontology revealed that starvation-induced differentially expressed genes, which participate in different metabolic processes and, most interestingly, in determination of adult lifespan process. One of these up-regulated genes is *Thor*. *Thor *encodes a homolog of mammalian *PHAS1/4E-BP*, which in mammals is a critical regulator of a pathway that controls initiation of translation through binding eukaryotic initiation factor 4E (eIF4E) [52]. *Thor *plays an important role in the host immune defense [52] and is a target of *mTOR *[53]. *PHAS1/4E-BP *is involved in inhibiting translation due to its binding to the translation initiation factor 4E, so the up-regulation of *Thor *during starvation inhibits translation and cell growth. Similar changes may be due to oxidative stress [54]. Also, up-regulation was identified in response to starvation in previous studies [55,54], and its link with longevity is associated with *mTOR *and *MnSOD *mechanisms [56]. Differential expression of *Thor *in response to other investigated stresses was not observed.

Genes up-regulated under conditions of ionizing radiation from 144 to 864 Gy enrich 39 biological processes. Two biological processes, telomere maintenance and telomere capping, are enriched with over-expressed genes specific for all three radiation doses. Also, the processes such as DNA repair, response to DNA damage stimulus, double-strand break repair and others characteristic to the high dose radiation exposure are not enriched by differentially expressed genes; the genes, involved in these processes, for instance, *lig3 *(DNA ligase III), *rad50 *(DNA repair protein RAD50), *Ku80*, *Irbp *(Inverted repeat-binding protein), *agt *(O-6-alkylguanine-DNA alkyltransferase), *mre11 *(meiotic recombination 11), and *mus205 *(mutagen-sensitive 205) were up-regulated by a high dose of radiation.

Sperm individualization and mRNA processing biological processes were down-regulated by 360 and 864 Gy radiation. Radiation exposure of 360 Gy was followed by down-regulation of 12 biological processes including mating behavior, the Notch signaling pathway, and different catabolic processes. Notch signaling plays roles in many organism functions, and changes in this pathway have been previously identified under the action of ionizing radiation [57]. And finally, 10 biological processes were down-regulated by 864 Gy, including sperm competition and multicellular organism reproduction. Ionizing radiation can sterilize insects [58], so differential expression of genes related to reproduction is expected.

Gene Set Enrichment Analysis using KEGG pathways showed not only a large number of differentially expressed metabolism and molecular synthesis pathways, but also changes in biosynthesis and functions of folates. "One carbon pool by folate" and "folate biosynthesis" KEGG pathways are enriched by genes differentially expressed by all treatments except 864 Gy radiation. Folates are donors of single carbons and therefore participate in different processes from DNA methylation [59] to de novo purine synthesis [60], which may further be used for RNA and DNA synthesis, including DNA repair. Hence, changes in folate biosynthesis may be explained as reaction to starvation, cold shock or irradiation because of their need in RNA synthesis or reparation.

Our laboratory studied differential expression in response to small doses of radiation (20 cGy) and chemical agents, such as toluene, formaldehyde, and dioxin [10]. Toluene and dioxin differentially expressed genes of metabolism of xenobiotics by the cytochrome P450 pathway. The oxidation-reduction process was most differentially expressed in the current and previous studies. We observed protective changes, such as in the expression of proteasome, nucleotide and base excision repair, mismatch repair, ubiquitin-dependent proteolysis, heat shock proteins, and basal transcription factors. Some of these were observed for radiation, fungus, and starvation treatments. Previously, we showed differential expression of genes related to immune response, to small doses of radiation, dioxin, toluene, as well as differential expression to metabolic genes for all these treatments except formaldehyde [10].

## Conclusion

The effects of stresses of different types (cold shock, ionizing radiation, fungal infection, starvation) and diverse doses of these stresses were examined. At higher doses of all stress factors except hypothermia, we detected more pronounced deleterious effects than at low doses. At the molecular level, transcriptional changes can be divided into stress type-specific changes and general effects for all examined stresses. For example, the expression of DNA repair genes showed direct proportionality to the dose of ionizing radiation, which may reflect dose-dependent increase in DNA damage. The Turandot gene family, which regulates immune reactions, may be considered as a candidate for genes involved in general stress responses.

In addition, differential expression (up- and down-regulation) of metabolic pathways can be considered as general stress response mechanism because it was observed in response to various types and doses of stress except entomopathogenic fungus. The metabolic changes were more significant under the low stress dose conditions. The folate metabolic and biosynthetic pathways were up- and down-regulated in the cases of all examined stresses, which may reflect their key role in general stress responses. Thus, the studied stress factors deleteriously affect the organism's viability and change both general and stress-specific cellular mechanisms.

## Material and methods

### Drosophila melanogaster strains

Stress response was tested on male *Drosophila melanogaster Canton-S *wild-type line (#1, Bloomington *Drosophila *Stock Center, Bloomington, USA).

The following transgenic lines were used as GFP reporters of stress-response genes:

• *Defensin-GFP, Drosomycin-GFP, Metchnikowin-GFP *contain GFP-reporters of genes of antimicrobial peptides activated by the Toll-dependent signaling pathway. These genes are up-regulated by 20 cGy irradiation [18]. We thank Dr. Won-Jae Lee (Seoul National University, Seoul, Korea) for these lines.

• *D-GADD45-GFP *contains a GFP reporter of the DNA damage response gene D-GADD45. *D-GADD45 *gene knockout results in dysfunctionality of radiation-induced hormesis and adaptive response [24].

• *GstD1-GFP *contains a GFP reporter of the glutathione S-transferase gene *GstD1*, involved in protection against oxidative stress and detoxification of xenobiotics. The promoter region of *GstD1 *contains consensus binding motifs for stress response transcription factors *Nrf2 *and *FOXO *[2]. We thank Dr. Tower (University of Southern California, Los Angeles, USA) for this line.

• *Hsp22-GFP, Hsp70-GFP *contain GFP reporters of heat shock proteins 70 and 22. These reporters are up-regulated by aging, heat shock, hyperoxia, hydrogen peroxide, and high doses of ionizing irradiation [61]. We thank Dr. Tower (University of Southern California, Los Angeles, USA) for these lines.

• *Stat92E-GFP *contains GFP reporters of STAT92E activator involved in generic stress response (#26200, Bloomington *Drosophila *Stock Center, Bloomington, USA). JAK-STAT signaling pathway is up-regulated by 20 cGy ionizing radiation [10].

### Maintenance conditions

Ten pairs of flies per vial with nutrients were left for 24 hours for mating and egg production. One day old imagoes were anesthetized with carbon dioxide and separated into vials with nutrients for further experiments. Flies were maintained in 12 hour illumination and relative humidity of 60% on yeast medium at densities of 30 flies per Narrow Fly Vial (Genesee Scientific, USA). Binder, KBF720-ICH, 720 l (Binder, Germany) climat chamber was used to stabilize maintenance conditions.

### Treatment by fungus

Entomopathogenic fungus *Beauvaria bassiana *was received from the State Research Institute of Genetics and Selection of Industrial Microorganisms (strain F-145, «Genetika», Russia). A protocol of Lemaitre et al. was used for the infection procedure [62]. Five day-old males were placed into a Petri dish with *Beauvaria bassiana*, followed by 60 seconds of shaking. *Beauvaria bassiana *was cultivated on agar medium. A 3 day-old culture with minimal sporulation was used for weak infection, and a 12 day-old culture with active sporulation was used for strong infection. This resulted in at least 10 and 100 colony-forming units per individual (CFU), correspondingly. The same procedure, but without infection was used on control flies. Based on survival analysis, we considered 10 CFU as a low dose and 100 CFU as a high dose of fungal infection.

### Treatment by ionizing radiation

Five day-old males were exposed to gamma-radiation from ^137^Cs (Russia) 'Issledovatel' equipment (Russia) with a dose rate of 0.72 Gy/min. The exposure time was 3 h 20 min, 8 h 20 min, and 20 h 00 min; the absorbed dose 144, 360, and 864 Gy correspondingly. Thermoluminescent dosimeters 'DTU-1' with detectors 'DTG-4' ('Doza', Russia) were used to assess the dose. The same procedure, but without irradiation, was used on control flies.

The literature states that there is a high resistance of *Drosophila melanogaster *to ionizing radiation. Fifty percent lethality 2 days post-treatment (LD_50/2_) in wild-type 1 day-old *Canton-S *males was observed for a dose of 1238 Gy [13]. We thus used a small dose (144 Gy) that was approximately 10 times below LD_50/2_, a high dose that was close to LD_50/2 _but not lethal (864 Gy), and medium dose (360 Gy).

### Cold shock

Five day-old males were exposed to temperatures of -4°C, 0°C, and +4°C in a thermostat for 120 min. Post-treatment flies were maintained in standard conditions. The same procedure but at +25°C in the thermostat was used on control flies. The temperatures for cold stress were selected after analyzing the published data. We used three temperatures to study the effects of varying intensity of the impact: from low (+4°C) through moderate (0°C) to hard (-4°C) [63,64,19].

### Starvation

Five day-old males were starved for 16 h in containers with 5 ml of 3% agar medium. The same procedure, but with standard medium, was used on control flies. Post-treatment flies were maintained in standard conditions. Starvation during 16 h was selected because it does not sufficiently reduce amount of investigated flies, but it influences survival and locomotor activity.

### Survival analysis

Dead flies were counted daily. Survival functions were estimated using the Kaplan-Meier procedure and plotted as survival curves. Median and maximum (age of 90% mortality) lifespan were calculated. Survival analysis was carried out in two experimental replicates for ionizing radiation, starvation, and hyperthermia and in 3 replications for fungi. A total of 200-350 individuals were analyzed for each type of stress.

### Analysis of locomotor activity

Locomotor activity was measured with software-hardware equipment Locomotor Activity Monitor (TriKinetics Inc., USA). Spontaneous locomotor activity was defined as averaged number of sensor crosses in 3 min per 30 flies. Locomotor activity was assessed one day after exposure to stress and then every 5 days. Measurements were carried out until 30 flies were alive in each variant.

### Quantification of GFP reporter gene expression

GFP reporters were used to validate RNA sequencing data. Expression of GFP reporters was measured by level of GFP fluorescence. Post-treatment flies (10 males for each variant) were anesthetized and visualised by fluorescence microscope 'MIKMED-2 v.11' ('LOMO', Russia) and Olympus C7070-based (Olympus, Japan) video system.

GFP expression analysis was performed with ImageJ software (National Institutes of Health, USA). Corrected total cell fluorescence (CTCF) coefficient was calculated in pixels and presented in histograms. To calculate the CTCF, we used the following formula: CTCF = Integrated Density - (Area of selected object × Mean fluorescence of background readings) [65].

### Statistical analysis

Nonparametric methods were used for survival analysis, namely, a modified Kolmogorov-Smirnov test for survival function, the Mantel-Cox test for median lifespan, and the Wang-Allison for maximum lifespan [66]. The χ^2 ^test was used for locomotor activity analysis. The T-test was used for statistical analysis of GFP reporter genes expression. Statistica v6.1 (StatSoft, USA) and R v2.15.1 (The R Foundation) were used for analysis.

### RNA Isolation and quality verification

Total RNA from 10 *Drosophila *imagoes for each sample was extracted using the miRNeasy Mini Kit (Qiagen, Germany) by following the manufacturer's instructions. Fluorescence quantitation was performed with Qubit^® ^2.0 Fluorometer (Invitrogen, USA). The quality of the total RNA was verified on Agilent 2100 Bioanalyzer using Agilent RNA 6000 Nano chip (Agilent Technologies, USA). RNA Integrity Number (RIN) value was ≥ 8 for each sample. There were two biological replicates of each sample.

### Total RNA sequencing sample preparation

Illumina TruSeq™ RNA Sample Preparation Kit (Low-Throughput protocol) was used to prepare samples for the mRNA sequencing libraries [67]. In summary, 2.5-3.0 μg of total RNA from each sample was used to purify the poly-A containing mRNA molecules by poly-T oligo-attached magnetic beads, with two rounds of purification. During the second elution of poly-A RNA, the RNA was also fragmented and primed for cDNA synthesis according to the manufacturer's protocol. cDNA synthesis from fragmented RNA and further cDNA conversion into double-stranded (ds) cDNA were performed by using a SuperScript Double-Stranded cDNA Synthesis kit (Invitrogen, USA). Ampure XP beads were used to separate ds cDNA from the second-strand reaction mix. The double-stranded cDNA was subjected to library preparation using the Illumina TruSeq™ RNA Sample Preparation Kit (Low-Throughput protocol) according to the manufacturer's protocol (Illumina, USA).

### Preparation of cDNA library

The construction of a cDNA library was performed according to the manufacturer's instructions using TruSeq™ RNA Sample Preparation Kit (Illumina, USA). 2.5 μg of high-quality RNA from each sample was used in purification and fragment mRNA procedures. The cleaved and primed mRNA fragments in turn were subjected to first-strand and second-strand cDNA synthesis. The generated double-stranded cDNA (ds cDNA) was further subjected to conversion of the overhangs resulting from fragmentation into blunt ends per the manufacturer's instructions. A single "A" nucleotide was added to the 3'-terminal ends of blunt fragments through adenylation reaction to prevent the ligating of them to one another during adapter ligation process. Then the multiple indexing adapters containing a single "T" nucleotide on the 3' end were complementarity ligated to the ends of the adenylated ds cDNA required for hybridization of cDNA library onto a flow cell. The in-line controls for tracking the steps involved in converting ds cDNA into libraries were used. Selective enrichment of cDNA fragments that have adapters ligated on both ends was performed using Illumina PCR Primer Cocktail in 11 cycle PCR reaction according to manufacturer's protocol. The library fragments were purified with AMPure XP system (Beckman Coulter, USA) after each step of enzymatic reaction.

### Library validation

Library quantification was performed using a qPCR method as described in [10]. The quality of the libraries was checked on Agilent 2100 Bioanalyzer using a High Sensitivity DNA chip (Agilent Technologies, USA). The final library products were bands at approximately 260 bp.

### Sequencing procedure

Indexed cDNA libraries were normalized to 2nM and pooled together in equal volumes. The clustering of pooled cDNA libraries was performed on a cBot Cluster Generation System (Illumina, USA) according to the vendor's instructions. After cluster generation, the cDNA library was sequenced on a HiSeq™2000 platform (Illumina, USA) and 50 bp single reads were generated. Raw sequencing reads were obtained using Illumina HiSeq Analysis Software and stored in FASTQ format.

### NGS data processing

Reads were trimmed and then mapped to *Drosophila melanogaster *transcriptome assembly BDGP6.27 using Tophat2. Read counts for genes were calculated using the HTSeq-count tool and for transcripts - using coverageBed [68]. The identification of differentially expressed genes by comparing experimental and control samples for each treatment was done using the R package DSS [69]. False discovery rates (FDR) were derived from p-values after Benjamini-Hochberg adjustment for multiple testing. Expression alterations with FDR < 0.05 were considered as statistically significant. Significant differentially expressed gene lists (with FDRs and log fold changes) are performed in Table S1. The analysis of the overlap of these lists for different dose of each stress exposure and for different stresses was carried out using the R package VennDiagram [70]. Analysis of dose-dependent differential expression was performed using the generalized linear model (GLM) approach implemented in the edgeR Bioconductor package [71]. We used stress dose rank as a predictor, e.g. 1 - 144 Gy, 2 - 360 Gy and 3 - 854 Gy for ionizing radiation exposure stress. Read counts were normalized using the TMM (trimmed mean of M-values) method [72].

Gene set enrichment analysis (GSEA) was performed using PANTHER and GeneMania [73,74], interaction networks analysis was performed using STRING and GeneMania resources [75,74]. GeneOntology Biological Processes and KEGG pathway ontologies were used to define sets for GSEA. Up-regulated and down-regulated genes were processed separately. KEGG Orthology-Based Annotation System (KOBAS 2.0) [76] was used as the source of KEGG terms in two stages. Firstly, differentially expressed genes were annotated with putative pathways and diseases by mapping the gene to genes in KEGG GENES. Secondly, data from the first stage was used for enrichment of KEGG pathways. The genes from whole genome were used as the default background distribution in this experiment. The Fisher exact test was selected for analysis with the Benjamini-Hochberg method [77] for the follow up FDR correction. Expression alterations with FDR < 0.05 were considered as statistically significant.

## Competing interests

The authors declare that they have no competing interests.

## Authors' contributions

AM, MS, EP, DP, AD, ED, NZ, LS, SZ, AK wrote the manuscript text. AM, MS, EP, DP, AD, ED, IS, NZ, LS, SZ carried out the experiments. AM, SZ, GK, AS, DB, and AK carried out the bioinformatic analysis. AM and AK supervised the bioinformatic research and text of the manuscript. All authors read and approved the final manuscript.

## Supplementary Material

Additional file 1 **Table S1 **Significant gene lists (with FDRs and log fold changes) Click here for file

## Declarations

Publication of this article has been funded by the grant of the President of the Russian Federation for the state support of young Russian scientists.

This article has been published as part of *BMC Genomics *Volume 16 Supplement 13, 2015: Selected articles from the 7th International Young Scientists School "Systems Biology and Bioinformatics" (SBB'2015): Genomics. The full contents of the supplement are available online at http://www.biomedcentral.com/bmcgenomics/supplements/16/S13.
